# Supplementary material for: A comprehensive analysis of clinical, quality of life, and cost-effectiveness outcomes of key treatment options for benign prostatic hyperplasia
Source: PLoS One. 2022 Apr 15;17(4):e0266824. doi: 10.1371/journal.pone.0266824 (PMC9012364; doi:10.1371/journal.pone.0266824)
Supplement: S1 Table — Abbreviations: AE, adverse event; BPH, benign prostatic hyperplasia; CT, combination therapy; IPSS, International Prostate Symptom Score; NA, not applicable; PUL, prostatic urethral lift; PVP, photoselective vaporization of the prostate; TURP, transurethral resection of the prostate; WVTT, water vapor thermal therapy. #The utility score of PVP is not available. Since TURP and PVP are invasive surgical procedures, the utility score of TURP was applied for PVP. *The mean time to recovery assumptions were validated by the clinical expert as the data were not available in the literature. **The mean time to recovery of the AEs was retrieved from the Rezum II trial. (DOCX) [file pone.0266824.s001.docx]

S1 Table: Utility inputs

|  | **Utility** | **Reference** | **Mean Time to Recovery (Days)** |
| --- | --- | --- | --- |
| **Treatment** |  |  |  |
| CT | -0.03 | Ackerman et al. [1] | 365* |
| PUL | -0.03 | Ackerman et al. [1] | 30* |
| WVTT | -0.03 | Ackerman et al. [1] | 30* |
| PVP | -0.05^#^ | Ackerman et al. [1] | 30* |
| TURP | -0.05 | Ackerman et al. [1] | 30* |
| Catheterization | -0.05 | Chen et al. [2] | 0.9 for PUL [3]  3.4 for WVTT [4]  1.7 for PVP [5]  2.5 for TURP [5] |
| **Health states** |  |  |  |
| BPH Mild (IPSS 1-7) | 0.99 | Baladi et al. [6] | NA |
| BPH Moderate (IPSS 8-19) | 0.90 | Baladi et al. [6] | NA |
| BPH Severe (IPSS 20-35) | 0.79 | Baladi et al. [6] | NA |
| Death | 0.00 | Ackerman et al. [1] | NA |
| **Periprocedural AEs** |  |  |  |
| TUR syndrome | -0.17 | Ackerman et al. [1] | 3* |
| Transfusion | -0.05 | Assumption | 1* |
| Immediate acute urinary retention | -0.18 | Ackerman et al. [1] | 7* |
| **Short-term AEs** |  |  |  |
| Bladder spasm | -0.06 | Ackerman et al. [1] | 30* |
| Urinary retention | -0.18 | Ackerman et al. [1] | 30.7** |
| Urinary tract infection | -0.07 | Ackerman et al. [1] | 13.3** |
| Pelvic pain | -0.03 | Ackerman et al. [1] | 72.5** |
| Hematuria | -0.20 | Rognoni et al. [7] | 29.5** |
| Dysuria | -0.03 | Ackerman et al. [1] | 38.2** |
| Urinary urge incontinence | -0.20 | Ackerman et al. [1] | 30* |
| Frequency and urgency | -0.03 | Ackerman et al. [1] | 53.2** |
| Encrusted implants | -0.03 | Ackerman et al. [1] | 30* |
| Urethral strictures | -0.06 | Ackerman et al. [1] | 30* |
| Bladder neck contraction | -0.06 | Ackerman et al. [1] | 30* |
| **Long-term AEs** |  |  |  |
| Erectile dysfunction | -0.09 | Ackerman et al. [1] | 365* |
| Urinary incontinence | -0.20 | Ackerman et al. [1] | 365* |
| Abbreviations: AE, adverse event; BPH, benign prostatic hyperplasia; CT, combination therapy; IPSS, International Prostate Symptom Score; NA, not applicable; PUL, prostatic urethral lift; PVP, photoselective vaporization of the prostate; TURP, transurethral resection of the prostate; WVTT, water vapor thermal therapy  ^#^The utility score of PVP is not available. Since TURP and PVP are invasive surgical procedures, the utility score of TURP was applied for PVP.  *The mean time to recovery assumptions were validated by the clinical expert as the data were not available in the literature.  **The mean time to recovery of the AEs was retrieved from the Rezum II trial. | | | |

References

1. Ackerman S.J., Rein A.L., Blute M., et al. Cost effectiveness of microwave thermotherapy in patients with benign prostatic hyperplasia: Part I-methods. Urology. 2000;56(6):972-80.

2. Chen H.W., Bercik R.S., Werner E.F., Thung S.F. Cost-effectiveness of percutaneous tibial nerve stimulation versus extended release tolterodine for overactive bladder. J Urol. 2012;187(1):178-84.

3. Roehrborn C.G., Gange S.N., Shore N.D., et al. The prostatic urethral lift for the treatment of lower urinary tract symptoms associated with prostate enlargement due to benign prostatic hyperplasia: The L.I.F.T. study. J Urol. 2013;190(6):2161-7.

4. McVary K.T., Gange S.N., Gittelman M.C., et al. Minimally invasive prostate convective water vapor energy ablation: A multicenter, randomized, controlled study for the treatment of lower urinary tract symptoms secondary to benign prostatic hyperplasia. J Urol. 2016;195(5):1529-38.

5. Bachmann A., Tubaro A., Barber N., et al. 180-W XPS greenLight laser vaporisation versus transurethral resection of the prostate for the treatment of benign prostatic obstruction: 6-month safety and efficacy results of a European multicentre randomised trial--The GOLIATH study. Eur Urol. 2014;65(5):931-42.

6. Baladi J.F., Menon D., Otten N. An economic evaluation of finasteride for treatment of benign prostatic hyperplasia. Pharmacoeconomics. 1996;9(5):443-54.

7. Rognoni C., Tarricone R. Healthcare resource consumption for intermittent urinary catheterisation: Cost-effectiveness of hydrophilic catheters and budget impact analyses. BMJ Open. 2017;7(1):e012360.
