# Supplementary material for: A comprehensive analysis of clinical, quality of life, and cost-effectiveness outcomes of key treatment options for benign prostatic hyperplasia
Source: PLoS One. 2022 Apr 15;17(4):e0266824. doi: 10.1371/journal.pone.0266824 (PMC9012364; doi:10.1371/journal.pone.0266824)
Supplement: S2 Table — Abbreviations: AE, adverse event; CT, combination therapy; PUL, prostatic urethral lift; PVP, photoselective vaporization of the prostate; TURP, transurethral resection of the prostate; WVTT, water vapor thermal therapy. *No AE was identified from published literature; 0.0% was assumed. †AE rates varied in the time frame. The lowest and highest AE rates were listed. ‡The inputs were extrapolated from 2 years to 5 years. (DOCX) [file pone.0266824.s002.docx]

S2 Table: Time-specific AE rates

| Early/Late AEs | Early AEs  (0-3 months) | | | | | Early AEs  (4 months-1 year) | | | | | Late AEs  (13 months-5 years) | | | | |
| --- | --- | --- | --- | --- | --- | --- | --- | --- | --- | --- | --- | --- | --- | --- | --- |
| **Periprocedural AEs** | **CT** | **PUL** | **WVTT** | **PVP** | **TURP** | **CT** | **PUL** | **WVTT** | **PVP** | **TURP** | **CT** | **PUL** | **WVTT** | **PVP** | **TURP** |
| TUR syndrome | NA | NA | NA | NA | 1.4%[1] |  |  |  |  |  |  |  |  |  |  |
| Transfusions | 0.0%^*^ | 0.0%^*^ | 0.0%^*^ | 0.0%[2] | 0.7%[2] |  |  |  |  |  |  |  |  |  |  |
| Immediate acute urinary retention | 0.0%^*^ | 0.7%[3, 4] | 3.7%[5] | 5.0%[2] | 1.4%[2] |  |  |  |  |  |  |  |  |  |  |
| **Short-term AEs** | **CT** | **PUL** | **WVTT** | **PVP** | **TURP** | **CT** | **PUL** | **WVTT** | **PVP** | **TURP** | **CT** | **PUL** | **WVTT** | **PVP** | **TURP** |
| Bladder spasm | 0.0%^*^ | 3.6%[3, 4] | 0.0%^*^ | 0.0%^*^ | 0.0%^*^ | 0.0%^*^ | 0.2%[3, 4] | 0.0%[5] | 0.0%^*^ | 0.0%^*^ | 0.0%^*^ | 0.0%[3, 4] | 0.0%[5] | 0.0%^*^ | 0.0%^*^ |
| Urinary retention | 0.1%[6] | 0.7%[3, 4] | 3.7%[5] | 10.8%[7] | 9.9%[7] | 0.1%[6] | 0.2%[3, 4] | 0.0%[5] | 1.4%[7]  7.7%[2]^†^ | 0.0%[7]  6.6%[2]^†^ | 2.2%[6]^‡^ | 0.0[3, 4] | 0.0%[5] | 0.7%[7]^‡^ | 0.2%[7]^‡^ |
| Urinary tract infection | 0.0%[6] | 2.9%[3, 4] | 3.7%[5] | 18.0%[7] | 11.3%[7] | 0.0%[6] | 0.0%[3, 4] | 0.0%[5] | 1.4%[7] 12.5%[2]^†^ | 0.0%[7]  7.5%[2]^†^ | 0.2%[6]^‡^ | 0.0%[3, 4] | 0.0%[5] | 5.6%[7] | 0.0%[7] |
| Pelvic pain | 0.0%^*^ | 17.9%[3, 4] | 2.9%[5] | 0.0%^*^ | 0.0%^*^ | 0.0%^*^ | 0.5%[3, 4] | 0.0%[5] | 0.0% ^*^ | 0.0%^*^ | 0.0%^*^ | 0.0%[3, 4] | 0.0%[5] | 0.0%^*^ | 0.0%^*^ |
| Hematuria | NA | 25.7%[3, 4] | 11.8%[5] | 10.8%[7] | 15.5%[7] | NA | 0.2%[3, 4] | 0.0%[5] | 0.7%[7]  7.4%[2]^†^ | 0.0%[7]  10.3%[2]^†^ | NA | 0.0%[3, 4] | 0.0%[5] | 0.0%[7] | 0.0%[7] |
| Dysuria | 0.0%^*^ | 34.3%[3, 4] | 16.9%[5] | 0.0%^*^ | 0.0%^*^ | 0.0%^*^ | 0.2%[3, 4] | 0.2%[5] | 0.0%^*^ | 0.0%^*^ | 0.0%^*^ | 0.0%[3, 4] | 0.0%[5] | 0.0%^*^ | 0.0%^*^ |
| Urinary urge incontinence | 0.0%^*^ | 3.6%[3, 4] | 0.0%[5] | 0.0%^*^ | 0.0%[8] | 0.0%^*^ | 0.2%[3, 4] | 0.0%[5] | 0.0%^*^ | 0.0%^*^ | 0.0%^*^ | 0.0%[3, 4] | 0.0%[5] | 0.0%^*^ | 0.0%^*^ |
| Frequency and urgency | 0.0%^*^ | 7.1%[3, 4] | 5.9%[5] | 0.0%^*^ | 0.0%^*^ | 0.0%^*^ | 0.7%[3, 4] | 0.0%[5] | 0.0%^*^ | 0.0%^*^ | 0.0%^*^ | 0.0%[3, 4] | 0.0%[5] | 0.0%^*^ | 0.0%^*^ |
| Encrusted implants | NA | 1.8%[4] | NA | NA | NA | NA | 1.8%[4] | NA | NA | NA | NA | 0.0%[4] | NA | NA | NA |
| Urethral strictures | NA | 0.0%^*^ | 0.0%^*^ | 2.9%[7] | 4.2%[7] | NA | 0.0%^*^ | 0.0%^*^ | 2.9%[2] | 2.1%[7]  3.5%[2]^†^ | NA | 0.0%^*^ | 0.0%^*^ | 0.7%[7] | 0.0%[7] |
| Bladder neck contraction | NA | 0.0%^*^ | 0.0%^*^ | 0.0%^*^ | 0.0% ^*^ | 0.0% ^*^ | 0.0% ^*^ | 0.0% ^*^ | 0.0% ^*^ | 0.0% ^*^ | 0.0% ^*^ | 0.0% ^*^ | 0.0% ^*^ | 0.0% ^*^ | 0.0% ^*^ |
| **Long-term AEs** | **CT** | **PUL** | **WVTT** | **PVP** | **TURP** | **CT** | **PUL** | **WVTT** | **PVP** | **TURP** | **CT** | **PUL** | **WVTT** | **PVP** | **TURP** |
| Erectile dysfunction | 0.6%[6] | 0.0%[3] | 0.0%[9] | 2.6%[10] | 3.4%[10] | 0.6%[6] | 0.0%[3] | 0.0%[9] | 2.6%[10] | 3.4%[10] | 0.0%[6] | 0.0%[3] | 0.0%[9] | 0.0%[10] | 0.0%[10] |
| Urinary incontinence | 0.2%[6] | 1.3%[11] | 0.4%[12] | 10.8%[7] | 4.2%[7] | 0.2%[6] | 1.3%[11] | 0.4%[12] | 1.4%  7.7%[2]^†^ | 0.0%  2.8%[2]^†^ | 0.2%[6] | 1.3%[11] | 0.0%^*^ | 0.0%[7] | 0.0%[7] |
| S2 Table legend.  Abbreviations: AE, adverse event; CT, combination therapy; PUL, prostatic urethral lift; PVP, photoselective vaporization of the prostate; TURP, transurethral resection of the prostate; WVTT, water vapor thermal therapy  ^*^No AE was identified from published literature; 0.0% was assumed.  ^†^AE rates varied in the time frame. The lowest and highest AE rates were listed.  ^‡^The inputs were extrapolated from 2 years to 5 years. | | | | | | | | | | | | | | | |

References

1. Al-Ansari A., Younes N., Sampige V.P., et al. GreenLight HPS 120-W laser vaporization versus transurethral resection of the prostate for treatment of benign prostatic hyperplasia: A randomized clinical trial with midterm follow-up. Eur Urol. 2010;58(3):349-55.

2. Bachmann A., Tubaro A., Barber N., et al. 180-W XPS greenLight laser vaporisation versus transurethral resection of the prostate for the treatment of benign prostatic obstruction: 6-month safety and efficacy results of a European multicentre randomised trial--The GOLIATH study. Eur Urol. 2014;65(5):931-42.

3. Roehrborn C.G., Gange S.N., Shore N.D., et al. The prostatic urethral lift for the treatment of lower urinary tract symptoms associated with prostate enlargement due to benign prostatic hyperplasia: The L.I.F.T. study. J Urol. 2013;190(6):2161-7.

4. Roehrborn C.G., Barkin J., Gange S.N., et al. Five year results of the prospective randomized controlled prostatic urethral L.I.F.T. study. Can J Urol. 2017;24(3):8802-13.

5. McVary K.T., Gange S.N., Gittelman M.C., et al. Minimally invasive prostate convective water vapor energy ablation: A multicenter, randomized, controlled study for the treatment of lower urinary tract symptoms secondary to benign prostatic hyperplasia. J Urol. 2016;195(5):1529-38.

6. Roehrborn C.G., Siami P., Barkin J., et al. The effects of combination therapy with dutasteride and tamsulosin on clinical outcomes in men with symptomatic benign prostatic hyperplasia: 4-year results from the CombAT study. Eur Urol. 2010;57(1):123-31.

7. Thomas J.A., Tubaro A., Barber N., et al. A multicenter randomized noninferiority trial comparing GreenLight-XPS Laser vaporization of the prostate and transurethral resection of the prostate for the treatment of benign prostatic obstruction: Two-yr outcomes of the GOLIATH study. Eur Urol. 2016;69(1):94-102.

8. Eltabey M.A., Sherif H., Hussein A.A. Holmium laser enucleation versus transurethral resection of the prostate. Can J Urol. 2010;17(6):5447-52.

9. McVary K.T., Gittelman M.C., Goldberg K.A., et al. Final 5-year outcomes of the multicenter randomized sham-controlled trial of a water vapor thermal therapy for treatment of moderate to severe lower urinary tract symptoms secondary to benign prostatic hyperplasia. J Urol. 2021;206(3):715-724.

10. Leong J.Y., Patel A.S., Ramasamy R. Minimizing Sexual Dysfunction in BPH Surgery. Curr Sex Health Rep. 2019;11(3):190-200.

11. National Institute of Clinical Excellence (NICE). Lower urinary tract symptoms in men: Management. 2015. Available from: https://www.nice.org.uk/Guidance/CG97.

12. Dixon C.M., Cedano E.R., Pacik D., et al. Two-year results after convective radiofrequency water vapor thermal therapy of symptomatic benign prostatic hyperplasia. Res Rep Urol. 2016;8:207-16.
