# Supplementary material for: A comprehensive analysis of clinical, quality of life, and cost-effectiveness outcomes of key treatment options for benign prostatic hyperplasia
Source: PLoS One. 2022 Apr 15;17(4):e0266824. doi: 10.1371/journal.pone.0266824 (PMC9012364; doi:10.1371/journal.pone.0266824)
Supplement: S3 Table — (DOCX) [file pone.0266824.s003.docx]

S3 Table: Bibliography of 20 publications used in the network meta‐analysis

| Full-Text Citations | |
| --- | --- |
| 1 | Yoon C.J., Kim J.Y., Moon K.H., et al. Transurethral resection of the prostate with a bipolar tissue management system compared to conventional monopolar resectoscope: One-year outcome. Yonsei Med J. 2006;47(5):715-20. |
| 2 | Tugcu V., Tasci A.I., Sahin S., et al. Comparison of photoselective vaporization of the prostate and transurethral resection of the prostate: A prospective nonrandomized bicenter trial with 2-year follow-up. J Endourol. 2008;22(7):1519-25. |
| 3 | Autorino R., Damiano R., Di Lorenzo G., et al. Four-year outcome of a prospective randomised trial comparing bipolar plasmakinetic and monopolar transurethral resection of the prostate. Eur Urol. 2009;55(4):922-9. |
| 4 | Bouchier-Hayes D.M., Van Appledorn S., Bugeja P., et al. A randomized trial of photoselective vaporization of the prostate using the 80-W potassium-titanyl-phosphate laser vs transurethral prostatectomy, with a 1-year follow-up. BJU Int. 2010;105(7):964-9. |
| 5 | Elmansy H.M., Elzayat E., Elhilali M.M. Holmium laser ablation versus photoselective vaporization of prostate less than 60 cc: Long-term results of a randomized trial. J Urol. 2010;184(5):2023-8. |
| 6 | Singhania P., Nandini D., Sarita F., et al. Transurethral resection of prostate: A comparison of standard monopolar versus bipolar saline resection. Int Braz J Urol. 2010;36(2):183-9. |
| 7 | Akman T., Binbay M., Tekinarslan E., et al. Effects of bipolar and monopolar transurethral resection of the prostate on urinary and erectile function: A prospective randomized comparative study. BJU Int. 2013;111(1):129-36. |
| 8 | Hamouda A.G.M., Morsi G., Habib E., et al. A comparative study between holmium laser enucleation of the prostate and transurethral resection of the prostate: 12-month follow-up. J Clin Urol. 2014;7(2):99-104. |
| 9 | Bachmann A., Tubaro A., Barber N., et al. A European multicenter randomized noninferiority trial comparing GreenLight XPS laser vaporization and transurethral resection of the prostate for the treatment of benign prostatic obstruction: 12-month results of the GOLIATH study. J Urol. 2015;193(2):570-8 |
| 10 | Sonksen J., Barber N.J., Speakman M.J., et al. Prospective, randomized, multinational study of prostatic urethral lift versus transurethral resection of the prostate: 12-month results from the BPH6 study. Eur Urol. 2015;68(4):643-52. |
| 11 | Guo S., Muller G., Lehmann K., et al. The 80-W KTP GreenLight laser vaporization of the prostate versus transurethral resection of the prostate (TURP): Adjusted analysis of 5-year results of a prospective non-randomized bi-center study. Lasers Med Sci. 2015;30(3):1147-51. |
| 12 | Thomas J.A., Tubaro A., Barber N., et al. A multicenter randomized noninferiority trial comparing GreenLight-XPS Laser vaporization of the prostate and transurethral resection of the prostate for the treatment of benign prostatic obstruction: Two-yr outcomes of the GOLIATH study. Eur Urol. 2016;69(1):94-102. |
| 13 | Al-Rawashdah S.F., Pastore A.L., Salhi Y.A., et al. Prospective randomized study comparing monopolar with bipolar transurethral resection of prostate in benign prostatic obstruction: 36-month outcomes. J Urol. 2017;35(10):1595-601. |
| 14 | Purkait B., Sinha R.J., Srinivas K.S.A., et al. Outcome analysis of transurethral resection versus potassium titanyl phosphate-photo selective vaporization of the prostate for the treatment of benign prostatic hyperplasia; A randomized controlled trial with 4 years follow up. Turk J Urol. 2017;43(2):176-82. |
| 15 | Roehrborn C.G., Barkin J., Gange S.N., et al. Five year results of the prospective randomized controlled prostatic urethral L.I.F.T. study. Can J Urol. 2017;24(3):8802-13. |
| 16 | Gupta N., Rogers T., Holland B., et al. Three-year treatment outcomes of water vapor thermal therapy compared to doxazosin, finasteride and combination drug therapy in men with benign prostatic hyperplasia: Cohort data from the MTOPS trial. J Urol. 2018;200(2):405-13 |
| 17 | Kumar N., Vasudeva P., Kumar A., et al. Prospective randomized comparison of monopolar TURP, bipolar TURP and photoselective vaporization of the prostate in patients with benign prostatic obstruction: 36 months outcome. LUTS. 2018;10(1):17-20. |
| 18 | McVary K.T., Rogers T., Roehrborn C.G. Rezum water vapor thermal therapy for lower urinary tract symptoms associated with benign prostatic hyperplasia: 4-year results from randomized controlled study. Urology. 2019;126:171-9 |
| 19 | McVary K.T., Gittelman M.C., Goldberg K.A., et al. Final 5-year outcomes of the multicenter randomized sham-controlled trial of a water vapor thermal therapy for treatment of moderate to severe lower urinary tract symptoms secondary to benign prostatic hyperplasia. J Urol. 2021;206(3):715-724. |
| 20 | Otaola-Arca H., Alvarez-Ardura M., Molina-Escudero R., et al. A prospective randomized study comparing bipolar plasmakinetic transurethral resection of the prostate and monopolar transurethral resection of the prostate for the treatment of Benign Prostatic Hyperplasia: Efficacy, sexual function, quality of life, and complications. Int Braz J Urol. 2021;47(1):131-44. |
