# Supplementary material for: A comprehensive analysis of clinical, quality of life, and cost-effectiveness outcomes of key treatment options for benign prostatic hyperplasia
Source: PLoS One. 2022 Apr 15;17(4):e0266824. doi: 10.1371/journal.pone.0266824 (PMC9012364; doi:10.1371/journal.pone.0266824)
Supplement: S4 Table — Abbreviations: CT, combination therapy; ICER, incremental cost-effectiveness ratio; PUL, prostatic urethral lift; PVP, photoselective vaporization of the prostate; QALYs, quality-adjusted life years; TURP, transurethral resection of the prostate; WVTT, water vapor thermal therapy. Total costs were rounded to whole dollars and total QALYs were rounded to 3 decimal points. The exact total cost and QALY values were used to calculate all reported ICERs. (DOCX) [file pone.0266824.s004.docx]

S4 Table: Costs, QALYs, and ICER at 5 years for the five treatment options for men with moderate-to-severe lower urinary tract symptoms due to benign prostatic hyperplasia derived from the fixed-effects model

|  | **Generic CT** | **PUL** | **WVTT** | **PVP** | **TURP** |
| --- | --- | --- | --- | --- | --- |
| **Total cost** | $8,223 | $9,580 | $2,655 | $6,153 | $6,334 |
| **Total QALYs** | 4.113 | 4.124 | 4.189 | 4.219 | 4.230 |
| **Life years** | 4.799 | | | | |
| **Incremental cost relative to CT** | --- | $1,373 | -$5,566 | -$2,069 | -$1,888 |
| **Incremental QALYs relative to CT** | --- | 0.011 | 0.076 | 0.106 | 0.117 |
| **ICER versus CT** | --- | $126,619/QALY | dominates | dominates | dominates |
| S4 Table legend.  Abbreviations: CT, combination therapy; ICER, incremental cost-effectiveness ratio; PUL, prostatic urethral lift; PVP, photoselective vaporization of the prostate; QALYs, quality-adjusted life years; TURP, transurethral resection of the prostate; WVTT, water vapor thermal therapy  Total costs were rounded to whole dollars and total QALYs were rounded to 3 decimal points. The exact total cost and QALY values were used to calculate all reported ICERs. | | | | | |
