# Supplementary material for: A comprehensive analysis of clinical, quality of life, and cost-effectiveness outcomes of key treatment options for benign prostatic hyperplasia
Source: PLoS One. 2022 Apr 15;17(4):e0266824. doi: 10.1371/journal.pone.0266824 (PMC9012364; doi:10.1371/journal.pone.0266824)
Supplement: S5 Table — Abbreviations: CT, combination therapy; IPSS, International Prostate Symptom Score; PUL, prostatic urethral lift; PVP, photoselective vaporization of the prostate; SD, standard deviation; TURP, transurethral resection of the prostate; WVTT, water vapor thermal therapy. The analysis was conducted using an average IPSS of 22 as a baseline for all treatments. The IPSS changes of WVTT from years 1 to 5 were derived from 5-year Rezum II trial results [1]. The difference of the IPSS changes between WVTT and PUL, WVTT, PVP, and TURP, respectively, were derived from the network meta-analysis results and applied to each time point. (DOCX) [file pone.0266824.s005.docx]

S5 Table: IPSS change from baseline to year 5 from the fixed-effects network meta‐analysis model for use in the sensitivity analysis

|  | **CT** | | **PUL** | | **WVTT** | | **PVP** | | **TURP** | |
| --- | --- | --- | --- | --- | --- | --- | --- | --- | --- | --- |
|  | Mean | ±SD | Mean | ±SD | Mean | ±SD | Mean | ±SD | Mean | ±SD |
| **Baseline** | 22.0 | 4.8 | 22.0 | 4.8 | 22.0 | 4.8 | 22.0 | 4.8 | 22.0 | 4.8 |
| **3 months** | 11.0 | 6.4 | 11.1 | 6.4 | 10.6 | 6.4 | 10.2 | 6.4 | 10.2 | 6.4 |
| **6 months** | 10.6 | 6.2 | 10.7 | 6.2 | 9.8 | 6.2 | 9.0 | 6.2 | 8.9 | 6.2 |
| **1 year** | 11.9 | 6.7 | 12.2 | 6.8 | 10.3 | 6.7 | 8.6 | 6.9 | 8.5 | 6.9 |
| **2 years** | 11.8 | 6.2 | 12.1 | 6.3 | 10.2 | 6.2 | 8.5 | 6.4 | 8.4 | 6.4 |
| **3 years** | 12.1 | 6.1 | 12.4 | 6.2 | 10.5 | 6.1 | 8.8 | 6.3 | 8.7 | 6.3 |
| **4 years** | 13.0 | 7.4 | 13.3 | 7.5 | 11.4 | 7.4 | 9.7 | 7.6 | 9.6 | 7.6 |
| **5 years** | 12.7 | 7.8 | 13.0 | 7.8 | 11.1 | 7.8 | 9.4 | 8.0 | 9.3 | 8.0 |
| S5 Table legend.  Abbreviations: CT, combination therapy; IPSS, International Prostate Symptom Score; PUL, prostatic urethral lift; PVP, photoselective vaporization of the prostate; SD, standard deviation; TURP, transurethral resection of the prostate; WVTT, water vapor thermal therapy  The analysis was conducted using an average IPSS of 22 as a baseline for all treatments. The IPSS changes of WVTT from years 1 to 5 were derived from 5-year Rezum II trial results [1]. The difference of the IPSS changes between WVTT and PUL, WVTT, PVP, and TURP, respectively, were derived from the network meta‐analysis results and applied to each time point. | | | | | | | | | | |

Reference

1. McVary K.T., Gittelman M.C., Goldberg K.A., et al. Final 5-year outcomes of the multicenter randomized sham-controlled trial of a water vapor thermal therapy for treatment of moderate to severe lower urinary tract symptoms secondary to benign prostatic hyperplasia. J Urol. 2021;206(3):715-724.
