# Supplementary figures and images for: A comprehensive analysis of clinical, quality of life, and cost-effectiveness outcomes of key treatment options for benign prostatic hyperplasia
Source: PLoS One. 2022 Apr 15;17(4):e0266824. doi: 10.1371/journal.pone.0266824 (PMC9012364; doi:10.1371/journal.pone.0266824)

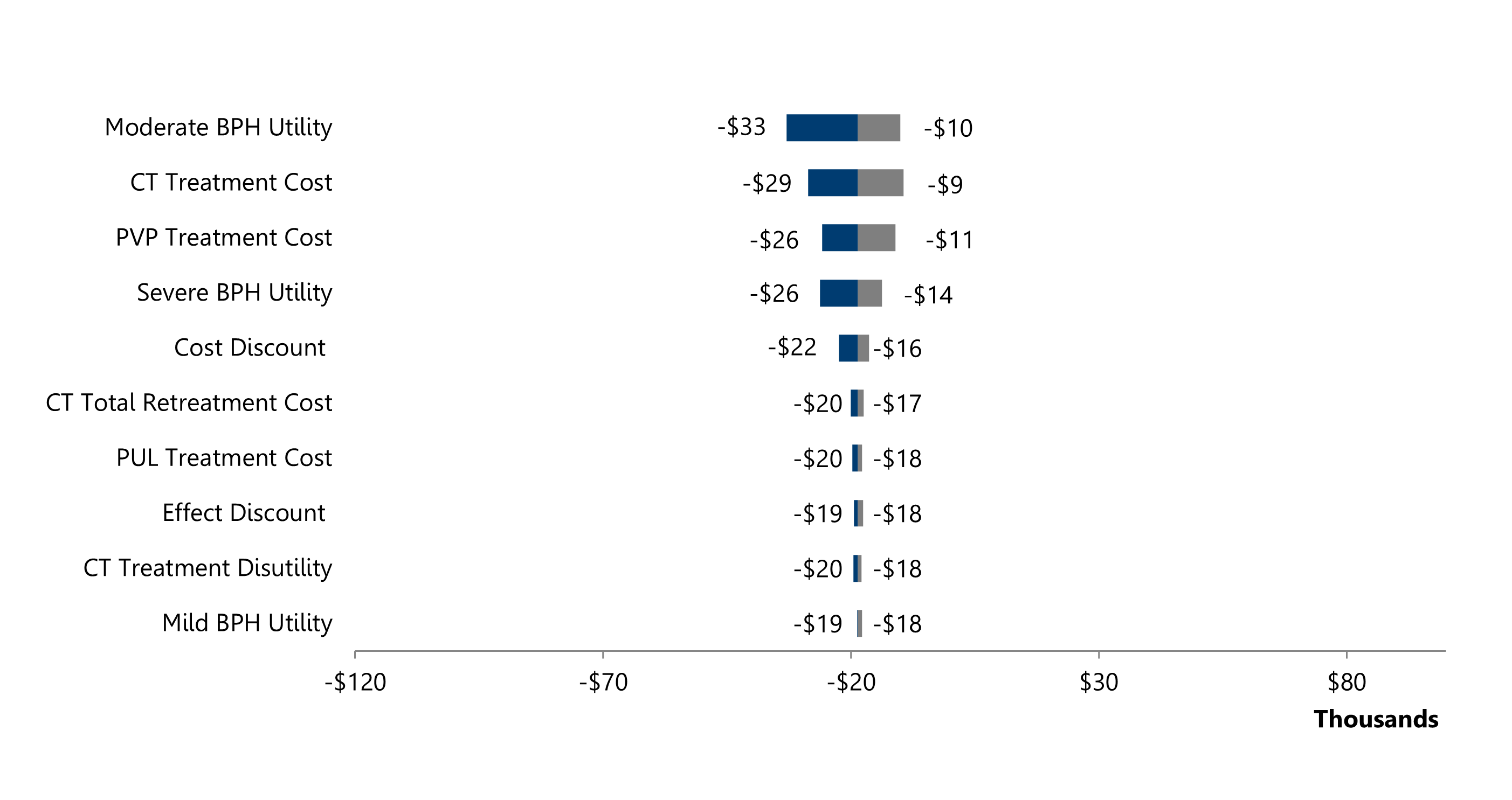

Supplement: S1 Fig — a. TURP vs CT. b. PVP vs CT. c. PUL vs CT. d. WVTT vs CT. Abbreviations: BPH, benign prostatic hyperplasia; CT, combination therapy; PUL, prostatic urethral lift; PVP, photoselective vaporization of the prostate; TURP, transurethral resection of the prostate; WVTT, water vapor thermal therapy. (ZIP) [file pone.0266824.s006.zip › S1b Fig_Accepted Version.tif]

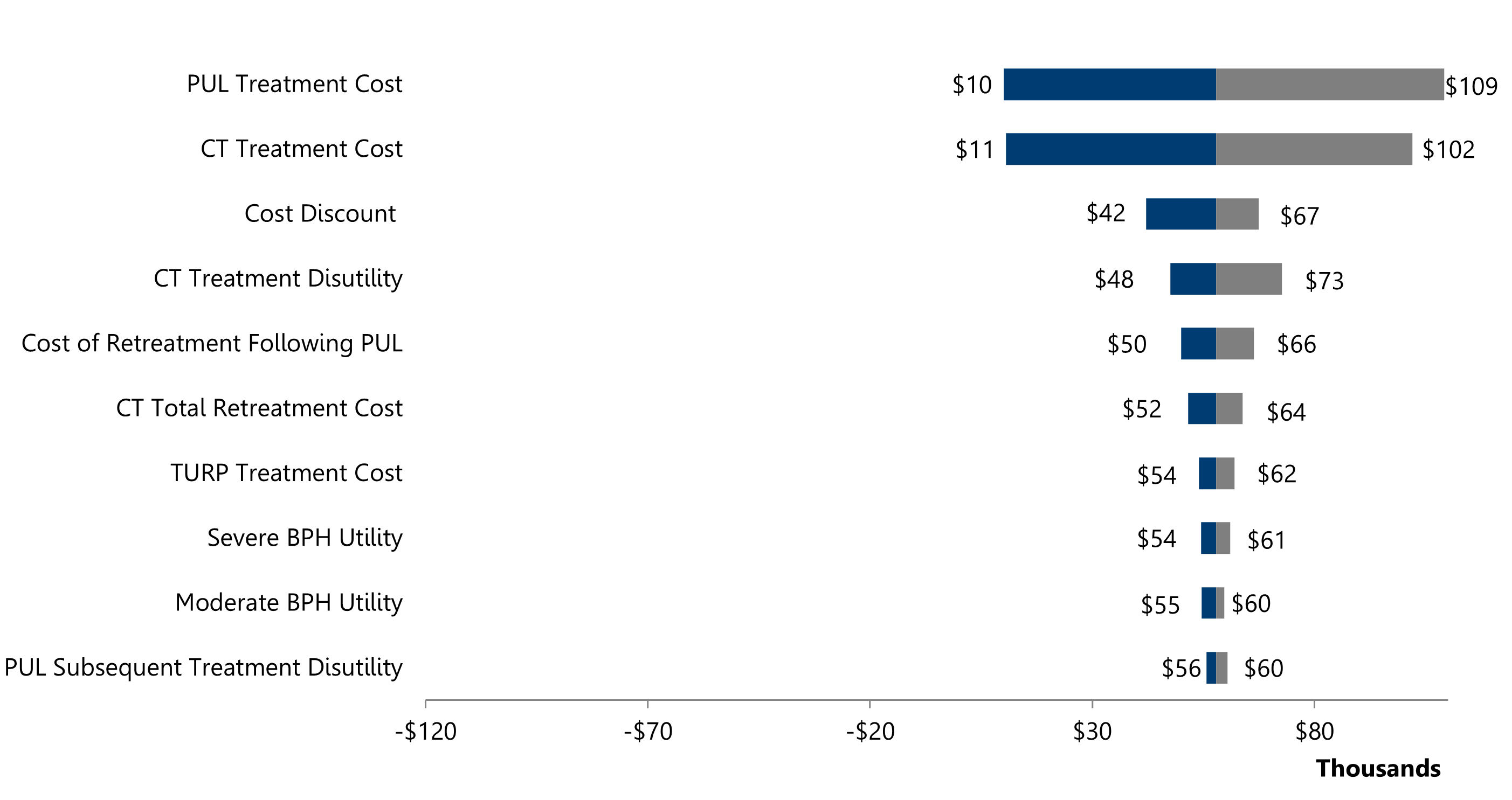

Supplement: S1 Fig — a. TURP vs CT. b. PVP vs CT. c. PUL vs CT. d. WVTT vs CT. Abbreviations: BPH, benign prostatic hyperplasia; CT, combination therapy; PUL, prostatic urethral lift; PVP, photoselective vaporization of the prostate; TURP, transurethral resection of the prostate; WVTT, water vapor thermal therapy. (ZIP) [file pone.0266824.s006.zip › S1c Fig_Accepted Version.tif]

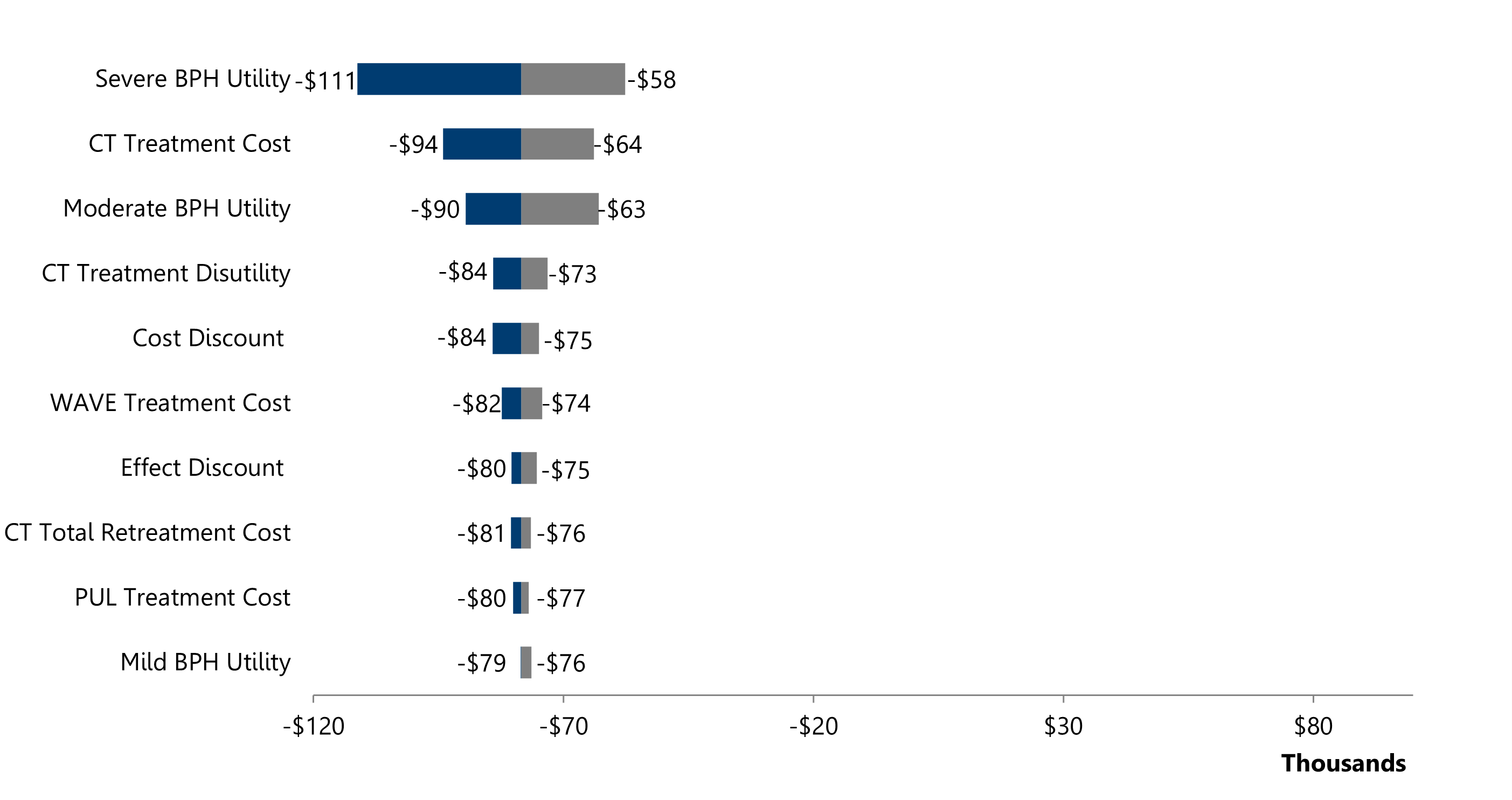

Supplement: S1 Fig — a. TURP vs CT. b. PVP vs CT. c. PUL vs CT. d. WVTT vs CT. Abbreviations: BPH, benign prostatic hyperplasia; CT, combination therapy; PUL, prostatic urethral lift; PVP, photoselective vaporization of the prostate; TURP, transurethral resection of the prostate; WVTT, water vapor thermal therapy. (ZIP) [file pone.0266824.s006.zip › S1d Fig_Accepted Version.tif]

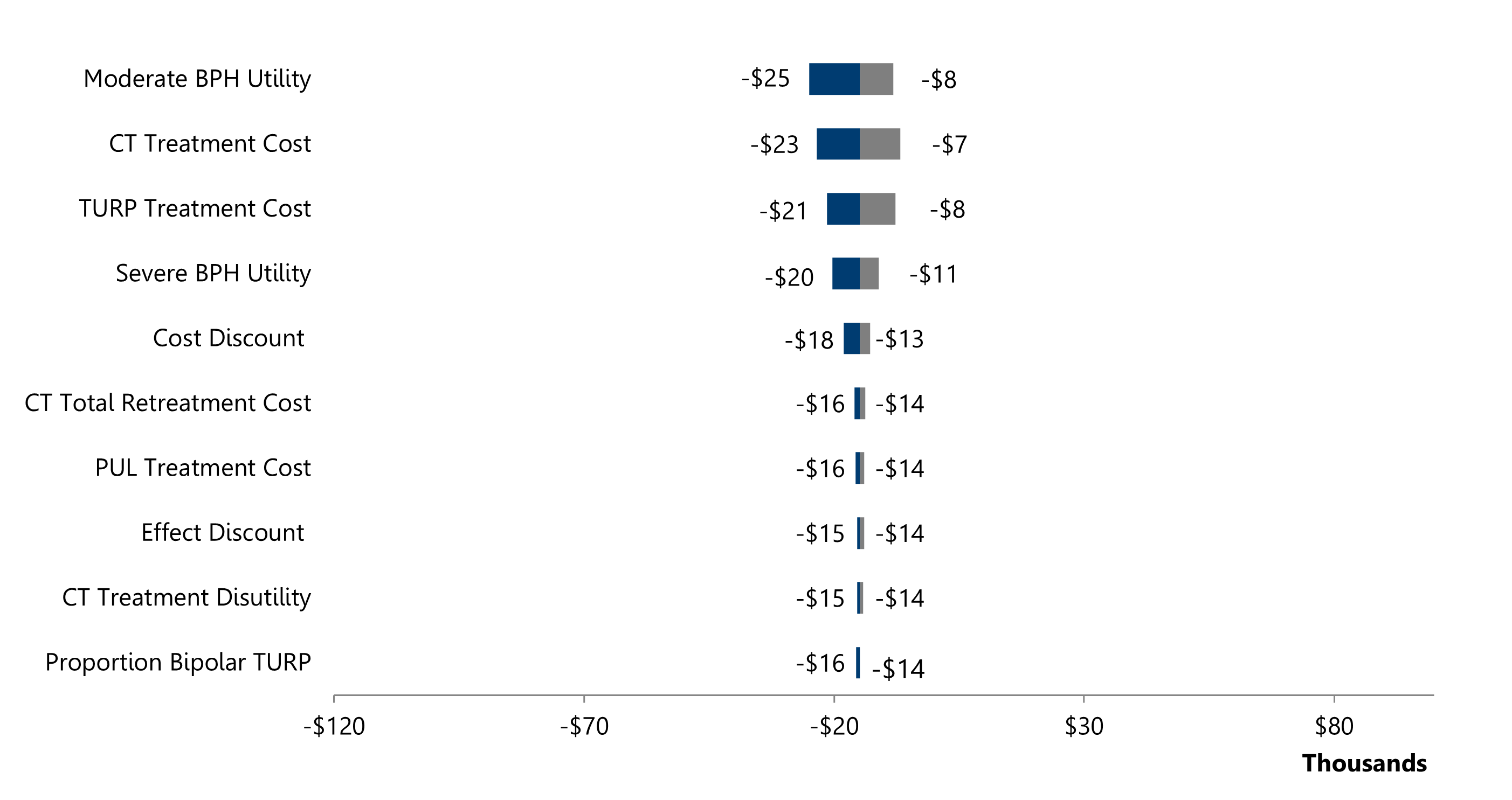

Supplement: S1 Fig — a. TURP vs CT. b. PVP vs CT. c. PUL vs CT. d. WVTT vs CT. Abbreviations: BPH, benign prostatic hyperplasia; CT, combination therapy; PUL, prostatic urethral lift; PVP, photoselective vaporization of the prostate; TURP, transurethral resection of the prostate; WVTT, water vapor thermal therapy. (ZIP) [file pone.0266824.s006.zip › S1a Fig_Accepted Version.tif]
