# Supplementary material for: A comprehensive analysis of clinical, quality of life, and cost-effectiveness outcomes of key treatment options for benign prostatic hyperplasia
Source: PLoS One. 2022 Apr 15;17(4):e0266824. doi: 10.1371/journal.pone.0266824 (PMC9012364; doi:10.1371/journal.pone.0266824)
Supplement: Attachment — Submitted filename: Rezum US CEA manuscript - Response to reviewers Final.docx [file pone.0266824.s007.docx]

**Reviewers’ Comments**

In your M&M you explain that you use the Rezum patient of ref 10 and the ‘model patient’. These patients had prostates 30-80gm and flowrates <15mL/s. I have two questions about that:

- Can you describe this model patient including the objective parameters that I have mentioned to provide the reader with more clarity, but more important: I think that many of the studies that you referred to have also included persons with (much) smaller prostates. The minimum of 30g has a good reason (with TURP as the comparator) because we do know that (TURP)resection/‘desobstruction’ in patients with small volumes hardly improves flowrates.

Response: Thank you very much for the great clinical insight. In our systematic review and meta-analysis, we used the inclusion criteria of men with prostate volumes ≤80 cm^3^. The criteria are documented on page 7 of the manuscript. From the systematic review, we found that the baseline mean prostate volumes ranged from 33.1 cm^3^ to 80.0 cm^3^ while baseline peak flow rates ranged from 4.5 mL/s to 9.9 mL/s. To clarify this point, we included the following statement in the result section of the manuscript on page 14 **“The baseline prostate volumes of patients in the 20 included publications ranged from 33.1 cm^3^ to 80.0 cm^3^ while their baseline peak flow rates ranged from 4.5 mL/s to 9.9 mL/s.”**

- Can you speculate/or conclude or comment on how this has affected costs. Is lowering the threshold to surgery which may be a consequence related to this cost-effective? (and are interventions not relatively more prone to re-intervention when done for patients without BPH and or with (only) other dysfunctions than bladder outflow obstruction?

Response: This is a really good observation. The retreatment rates are one of the key components that drive the total treatment costs. If lowering the threshold to surgery reduces the retreatment rates, changing the criteria for surgery would have an impact on the cost-effectiveness results. For BPH management, clinical guidelines including the 2021 American Urological Association (AUA) Guideline for BPH^1^ and the indication for use of treatment options include specific prostate volume details. The prostate volume details determine available treatment options for men with BPH. Therefore, lowering the threshold to surgery may not adhere to recommended clinical guidelines.

- Did you include (costs of) continued (LUT) medication in long term AE (not explicitly mentioned with ‘retreatment’ and or in the tables (these) mention a.e. symptoms but not treatment.

Response: For men who had recurring LUTS due to BPH symptoms post-MIST or surgical procedures, they could receive BPH medical therapy of generic tamsulosin 40 mg once daily long term. We have previously included the information in Table 1 on page 10. To better clarify this point in the manuscript, we have updated the following statement in the clinical input section of the manuscript on page 8 “Patients who experienced failed MIST or surgical procedures could either receive medical therapy **of generic tamsulosin 40 mg once daily** to manage their recurring symptoms, undergo the same treatment again, or undergo more invasive surgical procedures.”

This also brings me to your first sentence ‘Lower urinary tract symptoms (LUTS) attributed to benign prostatic hyperplasia (BPH)…’ the word ‘attributed to’ is not particularly cost saving (for all types of management including medication), or am I wrong? I ask you to consider commenting on this.

Response: “Lower urinary tract symptoms (LUTS) attributed to benign prostatic hyperplasia” is a common term used in the Urology field to describe the bothersome symptoms from an enlarged prostate gland. The same term is used in the AUA Guideline for the management of benign prostatic hyperplasia/ lower urinary tract symptoms.^1^ In this cost-effectiveness analysis context, only men with moderate-to-severe LUTS were considered given that if the conditions are not treated, the quality of life of these men would be negatively impacted. Men with anatomic enlargement of the prostate but without LUTS are not included as their first disease management is not a treatment but watchful waiting.

Last: You extrapolated follow up in your model. Can you better explain in the main text P5L94-96 what you did?

Response: Thank you for this comment. To clarify this point in the manuscript, we included the following statement in the clinical inputs section of the manuscript on page 8 “Since some AE rates were not available in the literature (S2 Table), we extrapolated the rates from 2 years to 5 years by applying the change of the AE rates between the second-last and last available quarter to subsequent cycles until the AE rates became 0%.”

Reference:

1. Lerner LB, McVary, KT, Barry MJ et al: Management of lower urinary tract symptoms attributed to benign prostatic hyperplasia: AUA Guideline part II, surgical evaluation and treatment. J Urol 2021; 206: 818.

**Journal Requirements**

When submitting your revision, we need you to address these additional requirements.

1. Please ensure that your manuscript meets PLOS ONE's style requirements, including those for file naming. The PLOS ONE style templates can be found at 
<https://journals.plos.org/plosone/s/file?id=wjVg/PLOSOne_formatting_sample_main_body.pdf> and
<https://journals.plos.org/plosone/s/file?id=ba62/PLOSOne_formatting_sample_title_authors_affiliations.pdf>

Response: I have confirmed that the manuscript follows PLOS ONE's style requirements.

2. We note that the grant information you provided in the ‘Funding Information’ and ‘Financial Disclosure’ sections do not match. When you resubmit, please ensure that you provide the correct grant numbers for the awards you received for your study in the ‘Funding Information’ section.

Response: The funding information is “This study was funded by Boston Scientific. SR, SKB, and BJC are employees of Boston Scientific. KN, DG, and SF are employees of Certara Evidence & Access, a consulting company that is paid by Boston Scientific for the services render. BC, AME, and KTM were not compensated for their participation in this study.

3. Thank you for stating the following in the Competing Interests section: 
[B Chughtai is a paid clinical consultant for Boston Scientific, MedeonBio, Olympus, and Allergan as well as an investigator for Teleflex. S Rojanasarot, B Cutone, and S Bhattacharyya are employees at Boston Scientific. K Neeser, D Gultyaev, and S Fu are employees of Certara Evidence & Access, a consulting company that is paid by Boston Scientific for the services render. A El-Arabi has no disclosures. K McVary is a principal investigator for NxThera, NIDDK, Astellas, Boston Scientific, and Olympus. Drs Chughtai, El-Arabi, and McVary were not compensated for their participation in this study.] Please confirm that this does not alter your adherence to all PLOS ONE policies on sharing data and materials, by including the following statement: "This does not alter our adherence to PLOS ONE policies on sharing data and materials.” (as detailed online in our guide for authors <http://journals.plos.org/plosone/s/competing-interests>).  If there are restrictions on sharing of data and/or materials, please state these. Please note that we cannot proceed with consideration of your article until this information has been declared. Please include your updated Competing Interests statement in your cover letter; we will change the online submission form on your behalf.

Response: I have included the updated competing interest statement in the cover letter.

4. In your Data Availability statement, you have not specified where the minimal data set underlying the results described in your manuscript can be found. PLOS defines a study's minimal data set as the underlying data used to reach the conclusions drawn in the manuscript and any additional data required to replicate the reported study findings in their entirety. All PLOS journals require that the minimal data set be made fully available. For more information about our data policy, please see <http://journals.plos.org/plosone/s/data-availability>.

Upon re-submitting your revised manuscript, please upload your study’s minimal underlying data set as either Supporting Information files or to a stable, public repository and include the relevant URLs, DOIs, or accession numbers within your revised cover letter. For a list of acceptable repositories, please see <http://journals.plos.org/plosone/s/data-availability#loc-recommended-repositories>. Any potentially identifying patient information must be fully anonymized.

Important: If there are ethical or legal restrictions to sharing your data publicly, please explain these restrictions in detail. Please see our guidelines for more information on what we consider unacceptable restrictions to publicly sharing data: <http://journals.plos.org/plosone/s/data-availability#loc-unacceptable-data-access-restrictions>. Note that it is not acceptable for the authors to be the sole named individuals responsible for ensuring data access.

We will update your Data Availability statement to reflect the information you provide in your cover letter.

Response: I have included the updated data availability statement in the cover letter. The statement is “All relevant data are within the manuscript and its Supporting Information files. Specifically, adverse event rates used in the model from different time points are included in S2 Table. Utility inputs are presented in S1 Table. All cost model inputs, including procedural, adverse event, retreatment and follow-up care costs, are in listed in Table 1. Total costs at 1 year and 5 years are summed of treatment, adverse events and follow up, and retreatment costs presented in Figure 2.”

5. Please review your reference list to ensure that it is complete and correct. If you have cited papers that have been retracted, please include the rationale for doing so in the manuscript text, or remove these references and replace them with relevant current references. Any changes to the reference list should be mentioned in the rebuttal letter that accompanies your revised manuscript. If you need to cite a retracted article, indicate the article’s retracted status in the References list and also include a citation and full reference for the retraction notice.

Response: I have reviewed and confirmed that reference list is appropriate.
