# Supplementary material for: A comprehensive analysis of clinical, quality of life, and cost-effectiveness outcomes of key treatment options for benign prostatic hyperplasia
Source: PLoS One. 2022 Apr 15;17(4):e0266824. doi: 10.1371/journal.pone.0266824 (PMC9012364; doi:10.1371/journal.pone.0266824)
Supplement: Attachment — Submitted filename: US CEA manuscript - Response to reviewers_Revision2_Final.docx [file pone.0266824.s008.docx]

**Journal Requirements:**

Please review your reference list to ensure that it is complete and correct. If you have cited papers that have been retracted, please include the rationale for doing so in the manuscript text, or remove these references and replace them with relevant current references. Any changes to the reference list should be mentioned in the rebuttal letter that accompanies your revised manuscript. If you need to cite a retracted article, indicate the article’s retracted status in the References list and also include a citation and full reference for the retraction notice.

Response: Many thanks again for checking the reference list. Tracking changes for the reference list cannot be done, so we have prepared a table below to document all changes to the reference list.

| Old reference | | New reference | |
| --- | --- | --- | --- |
| Number | Citation | Number | Explanation and new citation |
| 1 | McVary K. T., et al. Update on AUA guideline on the management of benign prostatic hyperplasia. The Journal of urology. 2011;185(5):1793-803. | NA | No replacement of this publication as the other publication included earlier is valid as a source for the statement included in the manuscript. All references in the manuscript and tables were re-indexed. |
| 2 | Welliver C., Feinstein L., Ward J.B., et al. Trends in lower urinary tract symptoms associated with benign prostatic hyperplasia, 2004 to 2013: the urologic diseases in America project. J Uro. 2020;203(1):171-8. | 1 | We cross-checked the BPH statistics in the first paragraph of the introduction section and have updated them accordingly. The same reference is used: Welliver C., Feinstein L., Ward J.B., et al. Trends in lower urinary tract symptoms associated with benign prostatic hyperplasia, 2004 to 2013: the urologic diseases in America project. J Uro. 2020;203(1):171-8. |
| 5 | Feinstein L., Matlaga B. Urologic diseases in America. Washington, DC: US Department of Health and Human Services, Public Health Service, National Institutes of Health, National Institute of Diabetes and Digestive and Kidney Diseases; 2018. | 4 | The page number was added to the original citation: Feinstein L., Matlaga B. Urologic diseases in America. Washington, DC: US Department of Health and Human Services, Public Health Service, National Institutes of Health, National Institute of Diabetes and Digestive and Kidney Diseases; 2018; NIH Publication No. 12-7865 **[pp. 78-81]**. |
| 6 | McVary K. T., Roehrborn C. G., Avins A. L., Barry M. J., Bruskewitz R. C., Donnell R. F., et al. American Urological Association Guideline: Management of Benign Prostatic Hyperplasia (BPH). American Urological Association Education and Research, Inc®. 2010 (Reviewed and validity confirmed 2014):1-496. | 5 | Lerner L.B., McVary K.T., Barry M. J., et al. Management of lower urinary tract symptoms attributed to benign prostatic hyperplasia: AUA Guideline part I-Initial work-up and medical management. J Urol. 2021;206(4):806-17. |
| 7 | Lerner L.B., McVary K.T., Barry M.J., et al. Management of lower urinary tract symptoms attributed to benign prostatic hyperplasia: AUA Guideline part II, surgical evaluation and treatment. J Urol 2021. https://doi.org/10.1097/  JU.0000000000002184. | 6 | Lerner L.B., McVary K.T., Barry M.J., et al. Management of lower urinary tract symptoms attributed to benign prostatic hyperplasia: AUA guideline part II-surgical evaluation and treatment. J Urol. **2021;206(4):818-26.** |

**Additional Editor Comments (if provided):**

Thank you for your revision and answers to the question. You have however not explained why ref 39 contradict your statement regarding prostate sizes. You have added 'tamulosin 40mg (which should be tamsilosin 40μg) but in fact patients receive 'CT' again (that should also include antimuscarinics). This should be included (likely with more than just one 'office visit' in 5y) unless you have good arguments not to include this in the calculations.

Response: Thank you very much for your feedback. We have broken down the response into four sections as follows:

**1. Prostate sizes (previous reference 39; current reference 38):** We would like to confirm that the 20 included publications used in the network meta-analysis (NMA) had baseline prostate volumes ranging from 33.1 cm^3^ to 80.0 cm^3^. Your understanding is correct that the baseline prostate volumes of patients from the previous reference 39^†^ were higher than 80.0 cm^3^. Because this study did not meet the inclusion criteria for the NMA, it was not included in the NMA to estimate the change in International Prostate Symptom Score (IPSS). Dixon et al. was included in the cost-effectiveness analysis as the study reported the adverse event rates due to urinary incontinence, which is the long-term adverse event outcome. If it was not included, the cost-effectiveness analysis would have underestimated the adverse event costs and reduced quality of life. Given that the mean and standard deviation of prostate volumes of patients in Dixon’s study were 48.6 ± 20.5 cm^3^, 95% of all prostate volumes from this study were within a range between 43.6 cm^3^ (lower 95% CI) and 53.5 cm^3^ (upper 95% CI).

- ^†^Dixon CM, et al. Two-year results after convective radiofrequency water vapor thermal therapy of symptomatic benign prostatic hyperplasia. Res Rep Urol. 2016;8:207-16.

To better clarify the references used in the NMA and the references used in other parts of the manuscript, we have included a new supplementary table (S3 Table) that lists all 20 publications used in the NMA. We believe the NMA bibliography should be added as a supplementary table because we currently have 38 references in the main manuscript. Please let us know if you agree with this approach.

**2. Tamsulosin 40 mg:** In our analysis, if the patients failed minimally invasive surgical treatments (MIST) or surgical procedures, they actually received tamsulosin 40 mg once daily to manage their recurring symptoms. The combination therapy (CT) of tamsulosin and dutasteride should not be used as a retreatment option since dutasteride, the 5-alpha reductase inhibitor used in this analysis, or other 5-alpha reductase inhibitors have a mechanism of action to reduce the formation of dihydrotestosterone, resulting in the reduction of the prostate size in benign prostatic hyperplasia. Given men have undergone MIST or surgical procedures earlier to remove their prostate, a monotherapy of tamsulosin 40 mg once daily would be more appropriate in treating the recurring lower urinary tract symptoms (LUTS).

**3. Inclusion of antimuscarinics:** We did not include antimuscarinics as a treatment option while we understand some failures could be due to underlying bladder issues. If antimuscarinics were included, they may lead to limited generalizability of the results given overactive bladder may co-exist with BPH in some men but not all men. Further, many of the trials exclude these men and thus we do not have clear clinical outcomes for these men.

**4. Number of office visits:** In our model, when patients were treated with CT as their index treatment, they had two office visits in the first year and from year two onward, they had one office visit per year for dose adjustment. The information is currently included in the clinical inputs section. For the patients who were retreated with medical therapy, the model incorporated the same annual office visit requirement as to those who underwent CT for dose adjustment. To clarify this point in the manuscript, we have incorporated the following statement in the clinical input section on **page 9** “The same office visit requirement was also applied to patients who received medical therapy as their retreatment.”

**Review Comments to the Author**

Please use the space provided to explain your answers to the questions above. You may also include additional comments for the author, including concerns about dual publication, research ethics, or publication ethics. (Please upload your review as an attachment if it exceeds 20,000 characters)

Reviewer #1: (No Response)

Reviewer #2: Well performed analysis of key metrics to consider in BPH therapy. Further comments on the applicability of this research to other parts of the world would be good to see.

Response: Many thanks again for the great feedback on the manuscript. We have added the following statement in the discussion section on **page 22**: “The results of this study could be used to inform decision-makers, including health technology assessment bodies outside of the US, when they assess the health economic value of each treatment option and adjust or establish reimbursed prices to ensure the balance between budget sustainability and patient access.”
