# Supplementary material for: A comprehensive analysis of clinical, quality of life, and cost-effectiveness outcomes of key treatment options for benign prostatic hyperplasia
Source: PLoS One. 2022 Apr 15;17(4):e0266824. doi: 10.1371/journal.pone.0266824 (PMC9012364; doi:10.1371/journal.pone.0266824)
Supplement: Attachment — Submitted filename: US CEA manuscript - Response to reviewers_Revision4.docx [file pone.0266824.s009.docx]

**Editor Comments**

I am very sorry but you still have not corrected the dosage of tamsilosin. Please connect with your co-authors!

Response: I have corrected the dosage of tamsulosin from 40 mg to 400 μg once daily. The co-authors and I cross-checked the dose and confirmed that 400 μg is the dose used in the US.

We note your Data Availability statement as follows: "No - some restrictions will apply; All relevant data are within the manuscript and its Supporting Information files. Specifically, adverse event rates used in the model from different time points are included in S2 Table. Utility inputs are presented in S1 Table. All cost model inputs, including procedural, adverse event, retreatment and follow-up care costs, are in listed in Table 1. Total costs at 1 year and 5 years are summed of treatment, adverse events and follow up, and retreatment costs presented in Figure 2."

PLOS journals require authors to make all data necessary to replicate their study’s findings publicly available without restriction at the time of publication. When specific legal or ethical restrictions prohibit public sharing of a data set, authors must indicate how others may obtain access to the data. (<https://journals.plos.org/plosone/s/data-availability>)

Before we can proceed, please address the following prompts:

a.) Please confirm if the data present in your manuscript and its Supporting Information files constitutes the minimal data set necessary to replicate your study's findings.

Response: I have confirmed that the data present in the manuscript and supporting information files constitutes the minimal data set necessary to replicate the study's findings.

b.) Please clarify what restrictions may apply to the data, and if these have prevented any of your minimal data set from being uploaded into the Supporting Information files.

Response: This manuscript and its supporting information files constitutes the minimal data set necessary to replicate the study’s findings. Other researchers could develop their own cost-effectiveness model using the data provided in the manuscript.
